# A C_**21**_-Steroidal Glycoside Isolated from the Roots of *Cynanchum auriculatum* Induces Cell Cycle Arrest and Apoptosis in Human Gastric Cancer SGC-7901 Cells

**DOI:** 10.1155/2013/180839

**Published:** 2013-12-19

**Authors:** Yi-Qi Wang, Shui-Juan Zhang, Hong Lu, Bo Yang, Liang-Fei Ye, Ru-Song Zhang

**Affiliations:** Department of Pharmacy, Zhejiang Chinese Medical University, Hangzhou, Zhejiang 310053, China

## Abstract

Caudatin 3-O-*β*-D-cymaropyranosyl-(1 → 4)-*β*-D-oleandropyranosyl-(1 → 4)-*β*-D-cymaropyranosyl-(1 → 4)-*β*-D-cymaropyranoside (CGII) is one of the C_21_-steroidal glycosides isolated from the roots of *Cynanchum auriculatum* ROYLE ex WIGHT. This study aimed to determine the cell growth, cell proliferation, and apoptotic cell death of human gastric cancer cells after CGII treatment. MTT assay was used to determine cell growth; fluorescence-activated cell sorting analysis was used to evaluate cell cycle distribution and apoptotic cell death. Immunoblotting was applied for measuring the expression of proteins involved in the cell cycle progression. The activities of caspase-3, -8, and -9 were detected by colorimetric caspase activity assays. CGII inhibited cell growth of human gastric cancer SGC-7901 cells in a concentration- and time-dependent manner. Treatment of SGC-7901 cells with CGII resulted in G1 phase cell cycle arrest, accompanied with decreased expression of cyclin D1 and cyclin-dependent kinases 4 and 6. CGII induced cell apoptosis and activated caspase-3, caspase-8, and caspase-9. In contrast, pan-caspase inhibitor z-VAD-fmk partially abolished the CGII-induced growth inhibition of SGC-7901 cells. In conclusion, CGII inhibits cell growth of human gastric cancer cells by inducing G1 phase cell cycle arrest and caspase-dependent apoptosis cascades.

## 1. Introduction 

Gastric cancer (GC) is a complex and enigmatic disorder noted for marked global variations in etiology, incidence, natural course, and management [1]. Systemic chemotherapy is currently used to manage advanced GC. However, due to drug toxicity and resistance, systemic therapy with classical cytotoxic drugs is poorly tolerated and ineffective [2]. Thus, there is an urgent need to identify new therapeutic agents for the treatment of GC in clinical practice.

**Figure 1:**
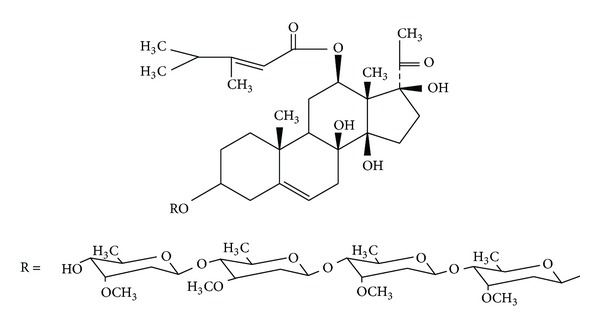
The chemical structure of CGII.

Natural products are very important compounds in the area of cancer chemotherapy due to their excellent pharmacological activities and low toxicity. There are numerous natural plants used for clinical cancer therapy in traditional Chinese medicine. The roots of *Cynanchum auriculatum* ROYLE ex WIGHT, known as “Baishouwu,” have been widely used for antiaging since ancient times. Its major components, C_21_-steroidal glycosides, are of considerable interest for pharmacological purpose. Previous studies have found that the C_21_-steroidal glycosides isolated from “Baishouwu” protect hepatocytes and neurons as well as gastric cells from toxicities [3–5]. Recent evidence has revealed the *in vitro* anticancer activity of some C_21_-steroidal glycosides [6–9]. Caudatin 3-O-**β**-D-cymaropyranosyl-(1 → 4)-**β**-D-oleandropyranosyl-(1 → 4)-**β**-D-cymaropyranosyl-(1 → 4)-**β**-D-cymaropyranoside (CGII) (Figure 1) is a C_21_-steroidal glycosides first isolated in 1995 [10]. However, there is a paucity of information on the pharmacological functions of this compound. In this study, we determined the cell growth, cell cycle distribution, and cell death pathways in human gastric cancer SGC-7901 cells after CGII treatment.

## 2. Materials and Methods

### 2.1. Extraction and Isolation of CGII

The roots of *C. auriculatum *(Gansu, Tianshui) were successively extracted with MeOH, CHCl_3_, and hexane. The hexane insoluble portion containing crude glycosides was subjected to column chromatography (SiO_2_; CHCl_2_/MeOH) to provide an active fraction containing 1.15% CGII. This fraction was further purified by HPLC (Waters, USA) to give CGII with a purity of more than 99%. The chemical structure of CGII (Figure 1) was elucidated by spectroscopic analysis including ^1^H NMR, ^13^C NMR, and HR-EI-MS.

### 2.2. Cells and Cell Culture

The human gastric cancer cell line SGC-7901 was obtained from the Shanghai Institute for Biological Sciences, Chinese Academy of Sciences, and cultured in RPMI 1640 (Gibco, USA) medium supplemented with 10% heat-inactivated fetal bovine serum (FBS, Gibco, USA), 100 U/mL penicillin, and 100 *μ*g/mL streptomycin at 37°C in humidified air containing 5% CO_2_.

### 2.3. MTT Assay

Cell proliferation was measured using the MTT (3-[4,5-dimethyl-2-yl]-2,5-diphenyl tetrazolium bromide, Sigma, USA) assay as described by van Meerloo et al. [11]. In brief, cells (3 × 10^3^) were seeded in each well of a 96-well plate and incubated at 37°C overnight. Cells were treated with CGII (0–80 *μ*M) or DMSO (0.1%, as a negative control) for 72 h to calculate IC_50_ (concentration required to inhibit the cell viability by 50%). In the experiment of observing the time-dependent properties, cells were treated with CGII (20 *μ*M or 40 *μ*M) for 24, 48, or 72 h. 20 *μ*L of MTT solution (5 g/L) was then added to each well, and the cells were incubated at 37°C for 4 h. The medium was discarded, and DMSO (150 *μ*L) was added. The optical density (OD) was measured at 570 nm with a microplate reader (Bio-Rad, USA). IC_50_ was calculated with NDST software (BioGuider Medicinal Technology, China).

### 2.4. LDH Activity Assay

Cell damage was measured by lactate dehydrogenase (LDH) assay using a colorimetric assay kit (JianCheng Bioengineering, China). SGC-7901 cells (3 × 10^3^) were seeded in each well of a 96-well plate and incubated at 37°C overnight. CGII (0–80 *μ*M) was added to treat cells for 72 h. Then LDH activity in the aspirated cell culture medium was examined by adding substrate. The OD value was measured at 450 nm and the LDH activity was calculated by comparing it with the standard.

### 2.5. Cell Cycle Analysis

SGC-7901 cells (1 × 10^6^) were seeded in a culture flask the day before drug treatment. Following treatment with CGII (20 Mm or 40 *μ*M) or DMSO (0.1%) for 24 h, the cells were trypsinized, washed twice with ice-cold phosphate-buffered saline (PBS), and fixed with ice-cold 70% ethanol in PBS at 4°C. After washing, the cell pellets were treated with 100 *μ*L of RNase A (1 g/L) at 37°C for 30 min and added to 900 *μ*L of staining buffer plus 20 *μ*L of propidium iodide (PI) (1 g/L) in the dark for 30 min. The samples were analyzed with a FACSCalibur flow cytometer and CellQuest analysis software (BD Biosciences, USA).

### 2.6. Western Blotting Assay

Cells were lysed with RIPA lysis buffer (Beyotime, China, 100 *μ*L) on ice. The protein concentration was determined by a BCA protein assay kit (KeyGEN, China). Equal amounts of proteins (60 *μ*g) from each group were separated by 12.5% SDS-PAGE and transferred to nitrocellulose membranes. Membranes were blocked with 5% nonfat milk in TBS (10 mM Tris and 100 mM NaCl) for 1 h and probed with the primary antibodies (Santa Cruz, USA) against cyclin-dependent kinase (CDK)-4, CDK6, cyclin D, and *β*-actin, respectively, followed by horseradish peroxidase-conjugated secondary antibody (Zhongshan, China) and ECL detection.

### 2.7. Apoptotic Cell Death Assay by FACS

Cell apoptosis was quantitatively determined by flow cytometry using an annexin V-FITC apoptosis detection kit (KeyGEN Biotech, China). Following the treatment, cells were harvested by trypsination, washed with PBS, and incubated with annexin V-FITC and PI at room temperature for 10 min in the dark. The stained cells were analyzed by an FACSCalibur flow cytometer and CellQuest analysis software.

### 2.8. Morphological Evaluation of Apoptotic Cells

SGC-7901 cells (1 × 10^5^) were seeded in 6-well plates the day before CGII treatment. After the treatment for 24 h, the cells were washed twice with PBS and observed with light microscopy (Olympus, Japan) or stained with acridine orange (5 *μ*L) for 10 min at room temperature in the dark and observed with a fluorescence microscope (Olympus, Japan).

### 2.9. Colorimetric Caspase-3, -8, and -9 Activity Assays

A colorimetric assay kit (KeyGEN, China) was used to detect the activities of caspase-3, caspase-8, and caspase-9. The cells (5 × 10^6^) were plated in a 75 cm^2^ flask the day before CGII treatment. After treatment for 24 h, the cells were washed twice with ice-cold PBS and lysed in lysis buffer on ice for 60 min. The protein concentration was determined by a BCA protein assay kit (KeyGEN, China). Protein samples of 50 *μ*g were used to test the caspase activities. The absorbance density was determined using a spectrophotometer at 400 nm (Amersham, USA).

### 2.10. Statistical Analysis

Data were expressed as mean ± SD. Values were analyzed by SPSS16.0 software for Windows, and the statistical significance of difference among the values was evaluated by one-way analysis of variance. A *P* value <0.05 was defined as significant.

## 3. Results

### 3.1. CGII Inhibits the Growth of SGC-7901 Cells

**Figure 2:**
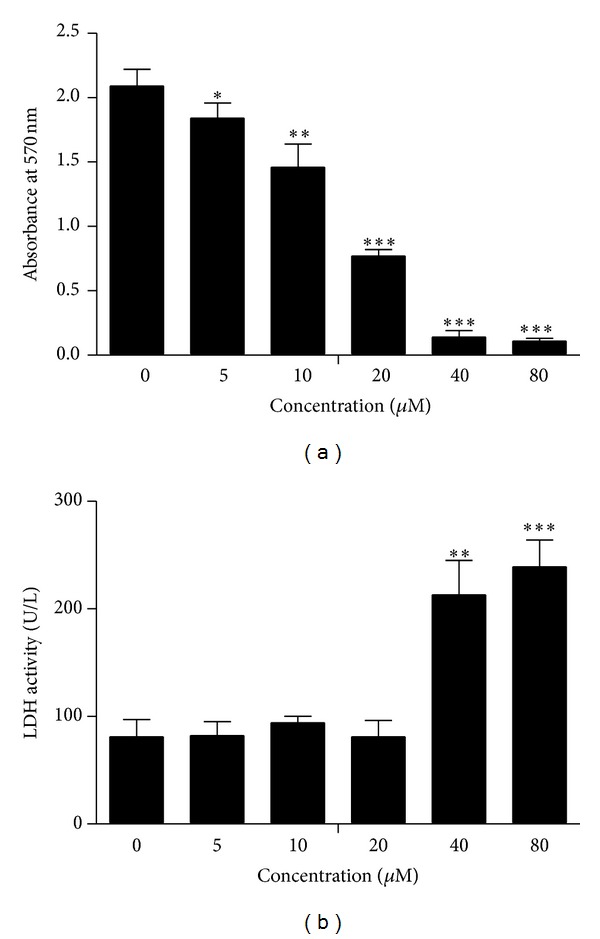
Concentration-dependent inhibitory effect of CGII on SGC-7901 cells. SGC-7901 cells were treated with CGII (0–80 *μ*M) for 72 h. An MTT assay was used to analyze cell proliferation. (a) LDH activity of cell culture medium was examined to evaluate cell damage. (b) The data were presented as mean ± SD, *n* = 3. *P < 0.05, **P < 0.01, ***P < 0.001, compared with the group without CGII treatment.

**Figure 3:**
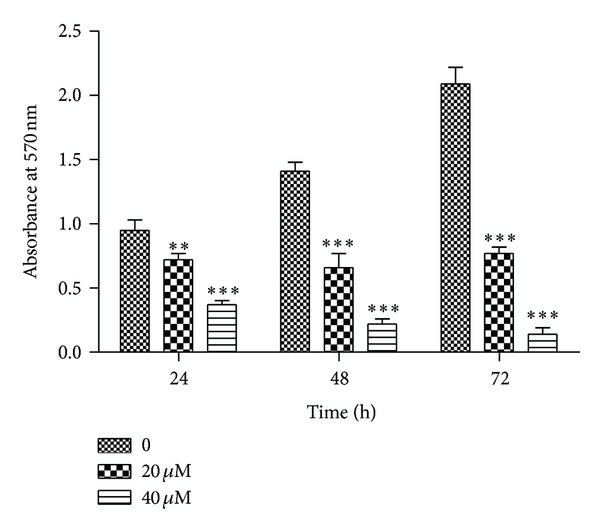
Time-dependent inhibitory effect of CGII on SGC-7901 cells. SGC-7901 cells were treated with CGII (0–40 *μ*M) for 24, 48, and 72 h, respectively. An MTT assay was used to analyze cell growth. The data were presented as mean ± SD, *n* = 3. **P < 0.01, ***P < 0.001, compared with the group without CGII treatment.

Cell growth is affected by both cell proliferation and viability. MTT assay was used to determine the effect of CGII on the proliferation of SGC-7901 cells and LDH activity of culture medium was measured to determine the cell damage induced by CGII. CGII at 0~80 *μ*M treated for 72 h concentration dependently inhibited the proliferation of SGC-7901 cells (Figure 2(a)). And 40 and 80 *μ*M CGII significantly increased the LDH activity in cell culture medium (*P* < 0.01, Figure 2(b)). As the IC_50_ of 72 h treatment of CGII in SGC-7901 cells was about 15 *μ*M, we next choose two concentrations of 20 and 40 *μ*M to further observe the time-dependent properties of CGII. Treatment with 20 *μ*M and 40 *μ*M CGII for 24 h resulted in 24.2% and 61.1% (*P* < 0.01) decreases of cell growth, respectively. Meanwhile, 53.2% and 84.4% (*P* < 0.001) decreases of cell growth were observed following treatment with 20 *μ*M and 40 *μ*M CGII for 48 h. And treatment with 20 *μ*M and 40 *μ*M CGII for 72 h led to 63.2% and 93.3% (*P* < 0.001) decreases of cell growth, respectively (Figure 3). Thus, CGII significantly inhibits cell growth of SGC-7901 cells in a concentration- and time-dependent manner.

### 3.2. CGII Induces G1 Phase Cell Cycle Arrest in SGC-7901 Cells

**Figure 4:**
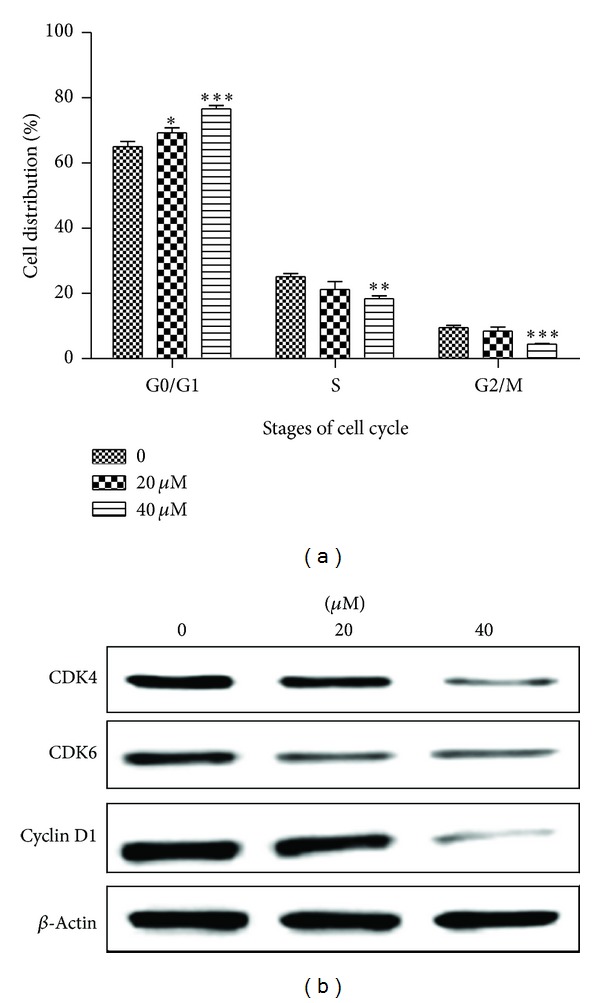
The effect of CGII on the cell cycle distribution and G1 regulatory CDKs and cyclins in SGC-7901 cells. The cells were treated with CGII at the indicated concentration for 24 h. (a) The cells were stained with PI and analyzed by flow cytometry. The data are presented as mean ± SD, *n* = 3. *P < 0.05, **P < 0.01, ***P < 0.001, compared with the group without CGII treatment. (b) SDS-PAGE and Western blot analysis.

To explore the underlying mechanism by which CGII inhibits cell growth of SGC-7901 cells, we determined the cell cycle progression of SGC-7901 cells after the treatment with CGII for 24 h. There were 65.1% of SGC-7901 cells in the G1 phase under normal growth conditions, while treatment of SGC-7901 cells with 20 *μ*M and 40 *μ*M CGII resulted in 69.3% (*P* < 0.05) and 76.6% (*P* < 0.001) of cells in the G1 phase of the cell cycle, respectively (Figure 4(a)). These results indicate that CGII induces G1 phase cell cycle arrest of SGC-7901 cells.

### 3.3. CGII Decreases the Protein Levels of CDK4/6 and Cyclin D1 in SGC-7901 Cells

It is well known that cyclin-dependent kinase (CDK) 4, CDK6, and cyclin D play crucial roles in the regulation of cell cycle progression from the G1 to S phase [12]. To determine whether CGII prevents G1 to S phase transition by downregulating the G1 CDKs, we measured the protein levels of CDK4, CDK6, and cyclin D. We found that treatment of SGC-7901 cells with CGII significantly decreased expressions of CDK4 and CDK6. Similarly, a significant reduction of cyclin D1 was observed with the treatment with 40 *μ*M CGII (Figure 4(b)), suggesting that CGII inhibits cell proliferation by partially downregulating the activity of CDK4 and CDK6.

### 3.4. CGII Induces Apoptosis in SGC-7901 Cells

**Figure 5:**
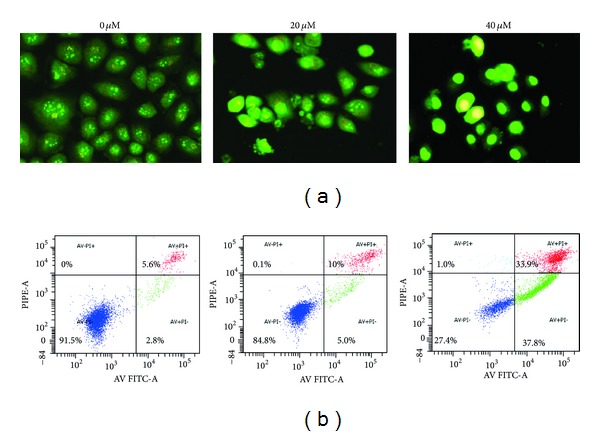
The apoptosis-inducing effect of CGII in SGC-7901 cells. The cells were treated with CGII at the indicated concentration for 24 h, stained with acridine orange, and observed with a fluorescence microscope magnification ×400 (a), or stained with annexin V-FITC and PI and analyzed by flow cytometry (b). The early apoptotic cells stained with green fluorescence are represented in the lower right (LR) quadrant of the FACS histogram, while the late necrosis cells stained with red-green fluorescence are represented in the upper right (UR) quadrant.

There is a large body of evidence showing that cellular distortion including nucleosomal fragmentation is associated with apoptosis induced by cytotoxic natural compounds [13]. To determine whether CGII treatment induces cell apoptosis, the fluorescent dye acridine orange, which binds to nuclear chromatin, was used to study the nuclear morphology changes in SGC-7901 cells. The fluorescent dye was observed to be distributed evenly in the nucleus of SGC-7901 cells in the DMSO control group. However, clear apoptotic characteristics, such as chromatin condensation, nuclear fragmentation, and apoptotic bodies, were observed in the cells exposed to CGII for 24 h. Moreover, the morphological changes became more pronounced with an increasing concentration of CGII (Figure 5(a)). Change in the cell membrane is another important marker of apoptosis; therefore, we analyzed the asymmetry and permeability of the cell membrane by annexin V-FITC and PI staining. As shown in Figure 5(b), the lower left quadrant contains viable cells, which exclude PI and are negative for annexin V staining. The lower right quadrant shows apoptotic cells, which still exclude PI but bind to green fluorescence labeled annexin V through phospholipids exposed on the cell surface of apoptotic cells. The upper quadrants represent necrotic cells that do not exclude PI and display red fluorescence. Treatment of SGC-7901 cells with CGII for 24 h significantly induced apoptosis and necrosis in a dose-dependent manner (Figure 5(b)). The percentages of total death cells (apoptosis and necrosis) were 2.8% in the cells treated with DMSO control, 15% in the cells treated with 20 *μ*M CGII, and 71.7% in cells treated with 40 *μ*M CGII, respectively.

### 3.5. CGII-Induced Apoptosis Is Mediated via Activation of Caspases in SGC-7901 Cells

**Figure 6:**
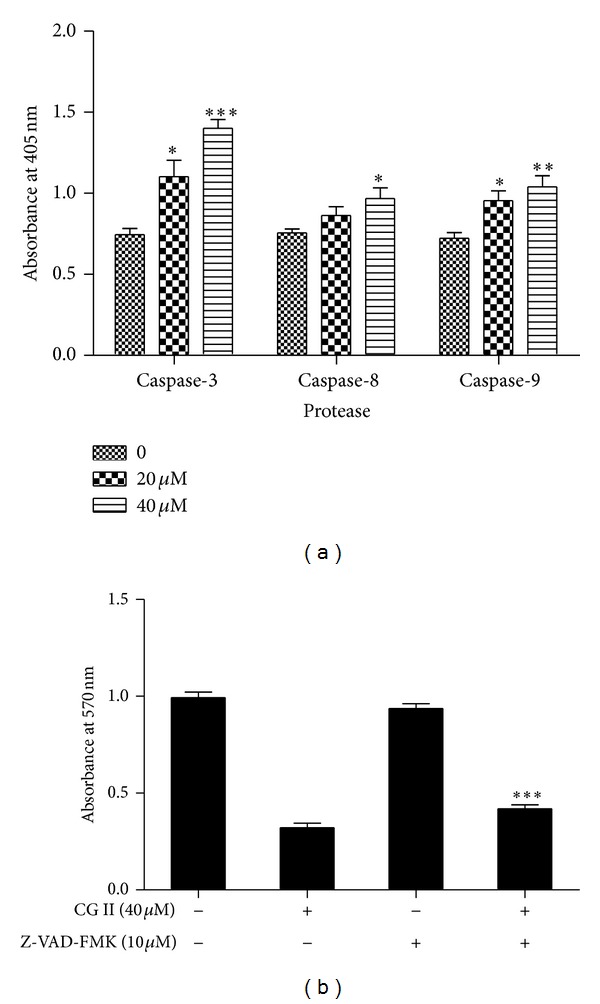
The effect of CGII on caspase activation in SGC-7901 cells. (a) The cells were treated with CGII (0–40 *μ*M) for 24 h. Total cell lysates were prepared and enzymatic activity in cell lysates was quantified by measuring the chromophores obtained from cleaved substrates. The data are presented as mean ± SD, *n* = 3. *P < 0.05, **P < 0.01, ***P < 0.001, compared with the group without CGII treatment. (b) The cells were treated with 40 *μ*M CGII or a mixture of 10 *μ*M z-VAD-fmk and 40 *μ*M CGII for 24 h, and an MTT assay was used to analyze cell growth. The data are presented as mean ± SD, *n* = 3. ***P < 0.001, compared with the group with CGII treatment alone.

Caspase-3 cleavage is the key event in the process of apoptosis and thus used as a marker of apoptosis induction. We next examined the effects of CGII on caspase-3 activity. As shown in Figure 6(a), treatment with 20 *μ*M and 40 *μ*M CGII for 24 h resulted in 1.5-fold (*P* < 0.05) and 1.9-fold (*P* < 0.001) increases of caspase-3 activity, respectively, compared with DMSO-treated cells. In a classical apoptotic cascade, caspase-3 can be activated by two pathways: an extrinsic pathway with the initiator caspase-8 or an intrinsic pathway in which the caspase activation cascade is mediated by caspase-9. To determine which pathway CGII activates caspase-3, we measured the activities of caspase-8 and -9 in SGC-7901 cells after CGII treatment. Treatment with 20 *μ*M and 40 *μ*M CGII for 24 h led to 1.1-fold and 1.3-fold (*P* < 0.05) increases in caspase-8 activity, and 1.3- (*P* < 0.05) and 1.4-fold (*P* < 0.01) increases in caspase-9 activity (Figure 6(a)), respectively. Thus, CGII activates caspase-3 through both the extrinsic and intrinsic pathways.

### 3.6. The Pan-Caspase Inhibitor z-VAD-fmk Suppresses CGII-Induced Cell Growth Inhibition in SGC-7901 Cells

To further support that the apoptosis induced by CGII is dependent on the caspase-3 pathway, we next determined whether the pan-caspase inhibitor z-VAD-fmk can promote the CGII-inhibited cell growth in SGC-7901 cells. Treatment with 40 *μ*M CGII for 24 h resulted in a 68% loss of cell viability, while cotreatment with z-VAD-fmk and CGII resulted in a 58% decrease of cell viability (Figure 6(b)). These results show that z-VAD-fmk partially reverses the growth inhibition by CGII in SGC-7901 cells.

## 4. Discussion

Deregulation of cell cycle checkpoints is a hallmark of cancer cells thus; inhibiting cell proliferation by inducing cell cycle arrest in cancer cells is an effective strategy for cancer therapy [14, 15]. In this study, we found that CGII obviously inhibited the cell growth of SGC-7901 cells *in vitro* by MTT assay. To determine whether the effect is related to cell cycle arrest, we examined the cell cycle changes after drug treatment. Our results demonstrated that treatment of SGC-7901 cells with CGII induced cell cycle arrest in the G1 phase, indicating that one of the mechanisms by which CGII inhibits the proliferation of SGC-7901 cells is through inhibition of cell cycle progression. Uncontrolled cell division depends on the sustained activation of CDKs [16, 17]. CDK4, CDK6, and cyclin D are the critical drivers for the G1 to S phase transition [18]. Our results demonstrated a marked decrease in the expression of CDK4, CDK6, and cyclin D in CGII-treated SGC-7901 cells. These data suggest that CGII induces G1 phase arrest through inhibition of the cyclin/CDK cascade in SGC-7901 cells.

Apart from inhibition of cell proliferation, apoptosis is another important event that anticancer drugs target [19]. In the previous study, we found different C_21_-steroidal glycosides with different cytotoxicity and apoptosis inducing properties [20, 21]. Therefore, we also explored whether the growth inhibition effect of CGII is related to apoptosis. We found that CGII treatment for only 24 h resulted in apparent apoptosis in SGC-7901 cells. Apoptotic cells are characterized by distinct morphological features such as cell shrinkage, loss of contact with neighboring cells, chromatin condensation, and formation of apoptotic bodies. To confirm the apoptosis-inducing effect of CGII, we observed the cell outline and nucleus using light and fluorescence microscopy and found that SGC-7901 cells treated with CGII exhibited typical apoptotic morphological features. Loss of phospholipids asymmetry of the plasma membrane is another typical feature in apoptosis [22]. We used annexin V-FITC staining to examine this apoptosis feature and PI staining was also used to discriminate necrosis cells. The results show that the 24 h treatment of CGII can increase the number of apoptosis cells in a concentration-dependent manner. However, an increase in necrosis cell number was also observed after drug treatment, which suggests that inducing apoptosis is not the only mechanism of the cytotoxicity of CGII.

There are two major pathways in mammalian cells leading to classical apoptosis: the caspase-8-initiated intrinsic and the caspase-9-dependent extrinsic pathways [23]. Caspase-3 is the common downstream molecule of caspase-8 and caspase-9. To elucidate the molecular mechanisms underlying the induction of apoptosis by CGII, we examined the activities of caspase-3, -8, and -9. To our surprise, CGII not only increased the activity of caspase-9 but also the activity of caspase-8. These results indicate that CGII induces caspase-dependent apoptosis through both the intrinsic and extrinsic pathways. Indeed, growing evidence has suggested that cytotoxic natural compounds have the ability to inhibit malignant cell growth by cell cycle arrest and by induction of apoptosis through both extrinsic and intrinsic pathways [24]. The induction of caspase-dependent apoptosis by CGII was further confirmed by the pan-caspase inhibitor z-VAD-fmk, which partially reduced the CGII-induced loss of cell viability in SGC-7901 cells. Apart from the classical caspase-dependent apoptosis pathways, caspase-independent apoptosis pathways exist [25]. Under conditions of caspase inhibition, the cell can utilize other default pathways towards self-destruction [26]. Our results showed that z-VAD-fmk only partially inhibited the viability loss in SGC-7901 cells treated with CGII, suggesting that CGII may induce apoptosis through some caspase-independent pathway under caspase inhibition conditions.

Though CGII can obviously inhibit SGC-7901 cell growth *in vitro*, the *in vivo* effect is unknown because the *in vivo* experiment cannot be performed at present due to the limited quantity of isolated CGII. However, we have evaluated the antitumor effect of total C_21_-steroidal glycosides from the roots of *Cynanchum auriculatum* ROYLE ex WIGHT in H_22_ and S_180_ tumor-bearing mice and tumor-bearing nude mice. The reduction of tumor weight can reach 65% compared with saline treated group (data is not reported). And in the future research, we will collect more compounds of C_21_-steroidal glycoside to perform *in vivo* experiment.

In summary, our results indicate that CGII significantly inhibits the cell growth and induces G1 phase arrest and apoptotic cell death of human gastric cancer SGC-7901 cells *in vitro*. The caspase-dependent pathways and caspase-independent pathways may participate in CGII-induced apoptosis of human gastric cancer cells. Our study paves the way for analysis of the association between the structure and activity of this type of compound.

## Abbreviations

GC: Gastric cancer
OD: Optical density
CDK: Cyclin-dependent kinase
PBS: Phosphate-buffered saline
PI: Propidium iodide.
